# Correlations of characteristics with tissue involvement in knee gouty arthritis: Magnetic resonance imaging analysis

**DOI:** 10.1016/j.heliyon.2024.e31888

**Published:** 2024-05-23

**Authors:** Qingshuai Wang, Bo Chen, Zhicheng Zhang, Xiongfeng Tang, Yingzhi Li

**Affiliations:** Department of Sports Medicine Arthroscopy, Second Hospital, Jilin University, Changchun, 130041, China

**Keywords:** Knee gouty arthritis, MRI, Monosodium urate deposition, Ligament, Meniscus, Cartilage

## Abstract

**Objective:**

This study investigates the MRI features of knee gouty arthritis (KGA), examines its relationship with the extent of tissue involvement, and assesses whether risk factors can predict KGA.

**Materials and methods:**

Patients diagnosed with KGA underwent MRI examinations, and two independent observers retrospectively analyzed data from 44 patients (49 knees). These patients were divided into mild and severe groups based on tissue involvement observed during arthroscopy. MRI features were summarized, and the intraclass correlation coefficient evaluated interobserver reproducibility. Single-factor analysis compared clinical indicators and MRI features between groups, while Cramer's V coefficient assessed correlations. Multivariate logistic regression identified predictors of tissue involvement extent, and a ROC curve evaluated diagnostic performance.

**Results:**

Among 49 knees, 18 had mild and 31 had severe tissue involvement. Key MRI features included ligament sketch-like changes, meniscal urate deposition, irregularly serrated cartilage changes, low-signal signs within joint effusion, synovial proliferation, Hoffa's fat pad synovitis, gouty tophi, bone erosion, and bone marrow edema. The interobserver reliability of the MRI features was good. Significant differences (P < 0.05) were observed between the groups for anterior cruciate ligament (ACL) sketch-like changes, Hoffa's fat pad synovitis, and gouty tophi. ACL sketch-like changes (r = 0.309), Hoffa's fat pad synovitis (r = 0.309), and gouty tophi (r = 0.408) were positively correlated with the extent of tissue involvement (P < 0.05). ACL sketch-like changes (OR = 9.019, 95 % CI: 1.364–61.880), Hoffa's fat pad synovitis (OR = 6.472, 95 % CI: 1.041–40.229), and gouty tophi (OR = 5.972, 95 % CI: 1.218–29.276) were identified as independent predictors of tissue involvement extent (P < 0.05). The area under the ROC curve was 0.862, with a sensitivity of 67.70 %, specificity of 94.40 %, and accuracy of 79.14 %.

**Conclusion:**

This comprehensive analysis of MRI features identifies ligament sketch-like changes, meniscal urate deposition, and low-signal signs within joint effusion as characteristic MRI manifestations of KGA. Irregular cartilage changes are valuable for differential diagnosis in young and middle-aged patients. ACL sketch-like changes, Hoffa's fat pad synovitis, and gouty tophi correlate with tissue involvement severity and are critical in predicting and assessing the extent of tissue involvement in KGA.

## Highlights

• This is the first comprehensive summary to analyze MRI features of KGA-affected tissues, and we found that ligament sketch-like changes, meniscal urate deposition changes, and point or diffuse low-signal signs within the joint effusion can be characteristic MRI manifestations for diagnosing KGA.
• MRI features can predict the extent of knee tissue damage in knee gouty arthritis, ACL sketch-like changes, Hoffa's fat pad synovitis, and gouty tophi are associated with the severity of knee joint tissue involvement.
• Arthroscopic verification of MRI imaging results can improve the MRI diagnosis of urate infiltration in bone, cartilage, ligaments, synovium, and other joint tissues of knee gouty arthritis.
• The future trend shifts from monitoring the location and severity of urate deposition to evaluating the extent of joint involvement and tissue damage.

## Abbreviations

**MRI**: Magnetic resonance imaging
**KGA**: Knee gouty arthritis
**ROC**: Receiver operating characteristic
**AUC**: area under the curve
**ACL**: Anterior cruciate ligament
**PCL**: Posterior cruciate ligament
**LCL**: Lateral collateral ligament
**MCL**: Medial collateral ligament
**MMAH**: Medial meniscus anterior horn
**MMB**: Medial meniscus body
**MMPH**: Medial meniscus posterior horn
**LMAH**: Lateral meniscus anterior horn
**LMB**: Lateral meniscus body
**LMPH**: Lateral meniscus posterior horn
**PJ**: Patellofemoral joint
**TJ**: Tibiofemoral joint
**MSU**: Monosodium urate
**DECT**: Dual-energy computed tomography
**BMI**: Body mass index
**UA**: uric acid
**CR**: creatinine
**ESR**: Erythrocyte sedimentation rate
**CRP**: C-reactive protein
**WBC**: white blood cell
**T1WI**: T1-weighted imaging
**PDWI-FS**: proton density-weighted fat-suppression
**ICC**: Intraclass Correlation Coefficient
**CI**: confidence interval
**OR**: odds ratio

## 1 Introduction

Knee gouty arthritis (KGA) is a prevalent inflammatory arthritis condition resulting from a disruption in purine metabolism and decreased uric acid excretion, which leads to the accumulation of monosodium urate (MSU) crystals [1]. These crystals cause inflammation in the knee joint's synovium, meniscus, and ligaments, leading to joint deformation and restricted mobility [2]. Clinically, symptoms such as joint pain, swelling, and instability are common, which can resemble those of meniscal or ligament injuries, complicating the diagnosis [3]. Recent epidemiological surveys indicate that the prevalence of KGA among adults in China has increased from 1 % to 1.3 % [4], showing a yearly upward trend. This increase highlights the need for accurate diagnosis and comprehensive evaluation of knee tissues, essential for effective treatment and improved patient outcomes. The diagnosis of KGA primarily relies on physicians' clinical experience, which can increase the risk of misdiagnosis. Thus, precise diagnosis and accurate assessment of the extent of knee joint tissue involvement are critical for proper management and possibly reversing the condition's progression, enhancing prognosis.

The international gold standard for diagnosing KGA involves detecting MSU crystals in joint fluid [5]. However, joint aspiration is invasive and can lead to complications. Studies show that synovial fluid detection has moderate sensitivity, with a 25 % chance of false negatives [6]. Consequently, non-invasive imaging techniques are crucial adjuncts to KGA diagnosis.

Ultrasound, dual-energy computed tomography (DECT), and magnetic resonance imaging (MRI) are key tools for diagnosing gout [7]. Ultrasound is cost-effective and valuable for detecting MSU crystals, gouty tophi, and related complications [8]. DECT provides quantitative analysis of urate crystal volume, important for disease assessment and subsequent follow-up [9]. However, both techniques have limitations in evaluating the extent of joint involvement in KGA. Ultrasound's accuracy can vary with the operator's skill and the precision of the equipment, leading to inconsistent results. DECT is less sensitive in the early stages of the disease [10,11]and may miss urate crystals or gouty tophi smaller than 2 mm, leading to false-negative results [12]. Additionally, DECT has limited capability in distinguishing soft tissues such as ligaments and menisci.

MRI is the preferred method for evaluating the knee joint and surrounding soft tissues. As a common clinical tool, MRI allows for detailed imaging of the joint, showcasing features such as gouty tophi, cartilage, meniscus, ligaments, and surrounding tissues. It is crucial in distinguishing KGA from other conditions [13,14]and assessing the extent of tissue impairment or inflammation within the joint. MRI not only detects gouty tophi and bone erosions effectively but also offers the benefit of being radiation-free. Moreover, MRI is crucial for monitoring early changes in tissues such as the intra-articular synovium, cartilage, ligaments, and menisci that are affected by urate crystal infiltration in the early stages of KGA. Consequently, MRI is essential for understanding disease progression and evaluating treatment options in KGA [7,15]. This tool can provide valuable imaging evidence for KGA diagnosis and treatment. However, the literature on the MRI features of knee gouty arthritis is limited, with most studies focusing on basic features such as gouty tophi and bone erosion. Comprehensive research on MRI features, particularly those affecting the cartilage, meniscus, or ligaments, remains scarce.

In a comparative study of diagnostic performance for KGA, arthroscopic examination demonstrated a sensitivity of 88.68 % and a specificity of 82.35 % [16]. Minimally invasive arthroscopy, compared to ultrasound, DECT, and MRI, shows superior diagnostic performance for KGA [[16], [17], [18]]. It can sensitively detect MSU crystals, provide comprehensive observations within the knee joint, and accurately assess the severity of MSU crystal deposition and tissue involvement. However, due to its invasive nature, minimally invasive arthroscopy is limited in its clinical application. This study focuses on summarizing and analyzing MRI characteristics of various knee joint tissues in KGA patients to enhance diagnostic accuracy. By integrating arthroscopy assessments, this research aims to identify risk factors predicting the extent of knee joint tissue involvement in KGA patients. Ultimately, this should help provide better imaging evidence for diagnosing and treating KGA, with the goal of achieving accurate diagnosis and improving outcomes.

## 2 Materials and methods

This research complied with the Helsinki Declaration guidelines. The Medical Ethics Committee of the Second Hospital of Jilin University granted approval, and informed consent requirements for the subjects were waived. The approval code is No. 2023136.

### 2.1 Participants

The study was carried out from June 2018 to June 2023 in the Department of Joint Surgery, Sports Medicine and Arthroscopy at the Second Hospital of Jilin University. The inclusion criteria for KGA patients were meeting the 2015 American College of Rheumatology/European League Against Rheumatism criteria for gout [19] and the presence of MSU crystal deposits under arthroscopy. Exclusion criteria included the lack of preoperative MRI and intraoperative arthroscopic images, other types of arthritis such as osteoarthritis, infectious arthritis, rheumatoid arthritis, etc., and fractures, tumors, severe cartilage injuries, or a history of previous surgery in the affected knee joint. Data on age, sex, disease duration, comorbidities, and body mass index (BMI) were collected. The laboratory parameters recorded included uric acid (UA), creatinine (CR), erythrocyte sedimentation rate (ESR), C-reactive protein (CRP), and white blood cell (WBC) counts.

**Fig. 1:**
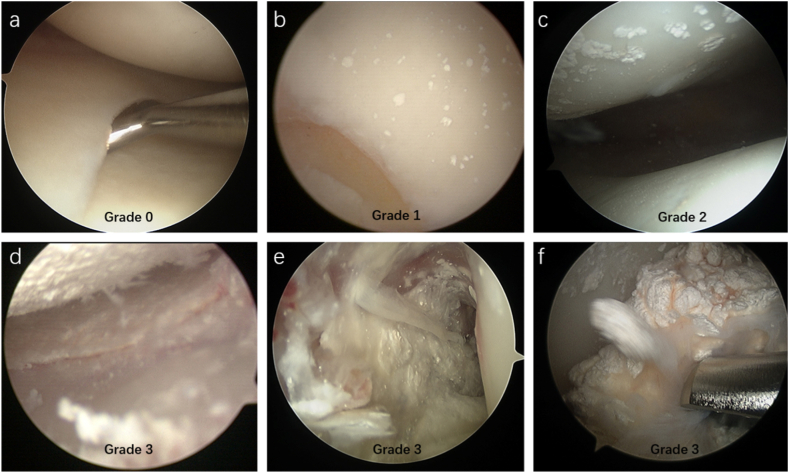
Arthroscopic grading of MSU crystal deposition in knee joints. (a) No significant deposition of MSU crystals. (b) Scattered distribution of MSU crystals. (c) < 50 % of the joint cartilage and soft tissue area showing multiple crystal depositions. (d–f) >50 % of the joint cartilage and soft tissue area exhibited multiple crystal depositions, including crystal erosion of the joint cartilage and subchondral bone, as well as evident tophi. Copyright © 2021 Gong et al.

A previous study [20] categorized MSU crystal deposition in the joint into four levels (Fig. 1): Grade 0(Fig. 1 a), Grade 1(Fig. 1 b), Grade 2(Fig. 1 c), and Grade 3(Fig. 1 d-f). All patients in this study had MSU crystal deposition (grade ≥1). Patients with Grade 3 deposition showed significantly worse knee joint tissue involvement and clinical symptoms than those with Grade 1 or 2. Thus, grades 1–2 were considered mild tissue involvement, while grade 3 was considered severe. Consequently, patients were divided into two groups based on severity of tissue involvement in the knee joint: a mild group (grades 1–2) and a severe group (grade 3).

### 2.2 MRI sequences

All patients underwent MRI using 3.0 T scanners from Philips. The imaging protocol included coronal, sagittal, and axial plane scans. The parameters were as follows: T1-weighted imaging (T1WI) in coronal and sagittal planes with an echo time of 10–30 ms, a repetition time of 300–700 ms, a 256 × 256 matrix, a 160 mm field of view, and a 3 mm slice thickness; proton density-weighted fat-suppression (PDWI-FS) sequences in coronal, sagittal, and axial planes with TE 19–40 ms, TR 1800–3500 ms, matrix 256 × 256, FOV 160 mm, and slice thickness of 3 mm.

### 2.3 Image analysis

MRI data analysis was conducted by two senior doctors with over 10 years of MRI diagnostic experience (one senior sports medicine physician and one senior radiologist). The main observational indicators were ligaments (anterior and posterior cruciate, and collateral), medial and lateral menisci, cartilage (in the patellofemoral and tibiofemoral joints), synovitis (encompassing joint effusion, synovial proliferation, and Hoffa's fat pad synovitis), gouty tophi, and bone marrow edema (in the patellofemoral joint/tibiofemoral joint). The MRI features of these components were comprehensively summarized in KGA. Patients were randomly grouped for image analysis, and outcomes were evaluated using a binary diagnostic approach. All image processing was done using RadiAnt DICOM Viewer 2022 software.

### 2.4 Statistical analysis

All statistical analyses were conducted using SPSS 26.0 software. Descriptive statistics presented the quantitative data for age, sex, and MRI features as mean ± standard deviation ($\bar{X}\pm s$). The intraclass correlation coefficient (ICC) estimates and their 95 % confidence interval (CI) [21,22]were calculated using a single-rating, absolute-agreement, two-way mixed-effects model to assess the test-retest reliability of each MRI feature. An ICC less than 0.40 was considered to indicate poor reliability, 0.40 to 0.60 as moderate reliability, 0.60 to 0.80 as good reliability, and above 0.80 as excellent reliability [23,24]. Normality tests assessed the data distribution. For normally distributed data, a *t*-test was used, while the Mann-Whitney *U* test was applied to data not meeting the normality assumption. Qualitative data are presented as percentages (%), and between-group differences were evaluated using the chi-square test. Correlation analysis utilized Cramer's V coefficient test. Statistically significant MRI features and clinically relevant variables associated with gout were included in the logistic regression analysis to identify independent predictive factors. The Receiver Operating Characteristic (ROC) curve evaluated the predictive value of the main influencing factors on the severity of KGA, including metrics such as the area under the curve (AUC), sensitivity, specificity, and accuracy. The Youden Index is calculated as Sensitivity minus (1 + Specificity), with its maximum value determining the optimal cutoff value of the ROC curve. A p-value less than 0.05 was considered statistically significant.

## 3 Results

### 3.1 Flowchart for participating in the study

This study initially screened 57 patients but excluded 13 due to various reasons, such as the absence of MRI or arthroscopic data (n = 9), presence of infectious arthritis (n = 3), and psoriatic arthritis (n = 1), leaving 44 patients (49 knees) in the study. The inclusion of these 5 additional cases did not introduce bias. Of the 49 knees, 18 (36.7 %) were categorized into the mild group, and 31 (63.3 %) into the severe group. A flowchart detailing patient selection and grouping is shown in Supplementary Fig. S1.

### 3.2 Analysis of the general conditions for the two groups of patients

Table 1 displays the comparative analysis results of basic clinical data, revealing no significant differences in sex, age, disease duration, UA concentration, ESR, etc., between the two groups (P > 0.05).

**Table 1:** Comparison of clinical indicators between the mild and severe KGA groups.

Characteristics	Mild group (n = 18)	Severe group (n = 31)	P value
Gender (Male)	17.94.4％	30.96.8％	–
Age (years)	37.28 ± 12.39	39.23 ± 9.95	–
Disease duration (months)	61.44 ± 54.79	78.61 ± 80.79	–
BMI (kg/m^2^)	26.77 ± 4.93	27.95 ± 3.17	–
CR (μmol/L)	77.11 ± 13.47	91.60 ± 25.95	–
UA (μmol/L)	520.28 ± 143.43	554.45 ± 138.90	–
WBC ( × 10^9^/L)	7.55 ± 3.00	8.35 ± 2.07	–
ESR (mm/h)	19.39 ± 15.12	43.35 ± 61.41	–
CRP (mg/L)	25.12 ± 50.93	28.81 ± 41.64	–

BMI, body mass index; CR, creatinine; UA, uric acid; WBC, white blood cell; ESR, erythrocyte sedimentation rate; CRP, C-reactive protein. *P < 0.05. “-" means P is greater than 0.05.

### 3.3 MRI features of knee gouty arthritis

The MRI study highlighted various manifestations in patients with KGA, including changes resembling ligament sketches, meniscal urate deposition, cartilage with irregular serration, point or diffuse low-signal signs within the joint effusion, synovial hyperplasia, Hoffa's fat pad synovitis, gouty tophi, bone erosion, and bone marrow edema.

### 3.4 Ligament sketch-like changes

**Fig. 2:**
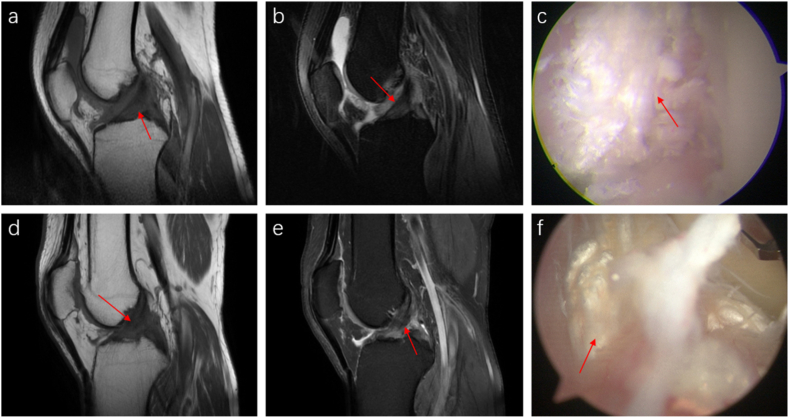
MRI and arthroscopic images of urate deposition in ligaments. A 46-year-old male patient with a 7-year history of KGA (a–c) exhibits uneven thickening in the anterior cruciate ligament (ACL) on sagittal T1-weighted (T1WI) (a) and proton density-weighted fat-suppressed (PDWI-FS) sequence (b). The T1WI reveals irregular patches with slightly low to moderate signal intensity inside within the ACL (red arrows), while the PDWI-FS sequence shows similar areas with moderate to slightly high signal intensity (red arrows). The ligament's morphology is irregular, showing sketch-like changes resembling injury signals, although the fiber course and angle remain largely normal. Arthroscopic examination (c) confirmed MSU crystal deposition on the ACL's surface. Another case involves a 30-year-old male patient with a 5-year history of KGA (d–e). The sagittal T1WI sequence (d) revealed similar irregular patches in both the anterior and posterior cruciate ligaments (red arrows). These patches showed moderate to slightly high signal intensity on the PDWI-FS sequence (red arrows) (e). Despite these changes, the overall course and angle of the ligaments were normal, and arthroscopic examination (f) shows irregular MSU crystals on the ligament surfaces.

On sagittal MRI, ligaments affected by MSU crystal deposition show uneven widening, primarily on the femoral side. These changes appear as irregular, patchy areas displaying slightly low to moderate T1 signal intensity and moderate to slightly high T2 signal intensity. The ligaments' orientation and angles generally remain normal. The disarray in the ligament fibers leads to blurred ligament outlines, resembling sketch lines. We refer to these alterations as “ligament sketch-like changes (Fig. 2 a, b, d, e). Arthroscopy confirmed irregular deposition of urate crystals within and on the surfaces of the ligaments and synovium (Fig. 2 c, f), accompanied by synovial hyperplasia or gouty nodules in adjacent areas.

**Fig. 3:**
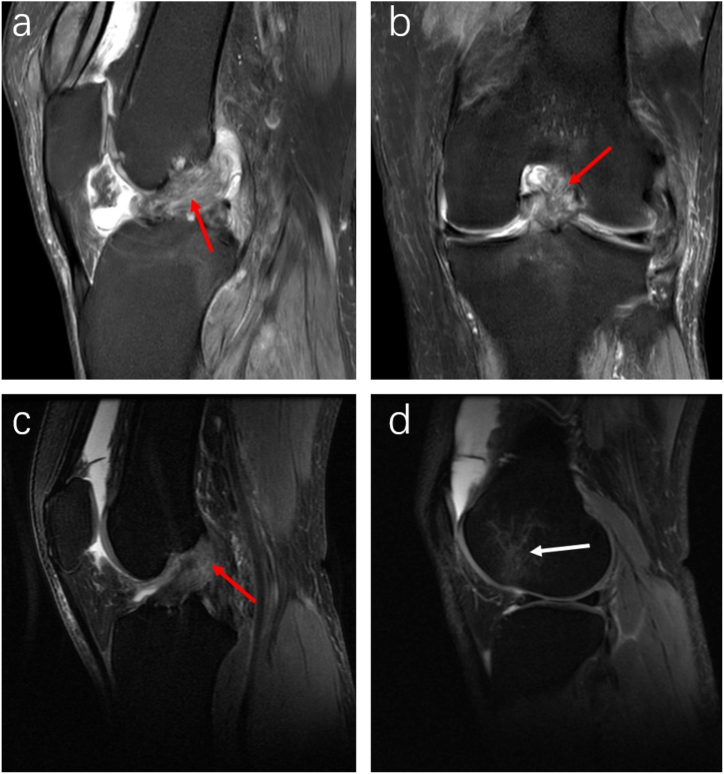
MRI features of severe ligament involvement (a, b). A 50-year-old male patient with a 23-year history of KGA exhibits complete disarray of ACL fibers on the sagittal and coronal PDWI-FS sequence. The sequence shows uneven signals and diffuse intermediate-to-high mixed signals marked by a red arrow, and “fibrous root-like” changes. MRI images indicate a combination of KGA and an ACL tear (c, d). Similarly, a 32-year-old male patient with KGA and an ACL tear displays a significantly curved and thickened ACL with increased internal signal intensity on the sagittal PDWI-FS sequence. The sequence reveals multiple patchy, slightly long T2 signal changes, and an irregular ACL femoral insertion point indicated by a red arrow. The fiber course direction and angle are diminished. Kissing contusions on the lateral femoral condyle are marked by a white arrow, presenting as a slightly high T2 signal with a diffuse distribution.

In a minority of cases with significant urate crystal deposits, ligaments may show extensive widening and completely disordered fibers, which present as diffuse mixed moderate to slightly high T2 signal intensity. However, the overall course of the ligament (Fig. 3 a, b) remains unaffected. MRI scans also indicated that patients with KGA who also have ligament tears show sketch-like changes in the ligaments, reduced ligament angles, and kissing lesion edema (Fig. 3 c, d).

### 3.5 Meniscal urate deposition changes

**Fig. 4:**
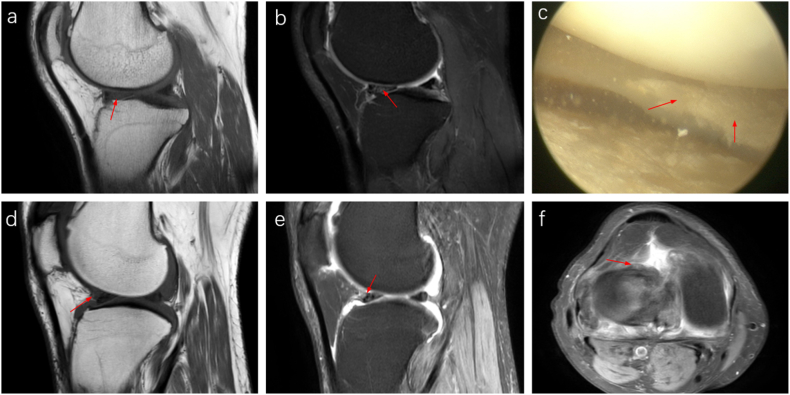
MRI and arthroscopic images of urate deposition in the meniscus. A 36-year-old male patient with a 13-year history of KGA (a, b). The sagittal T1WI (a) and PDWI-FS sequences (b) show a blurred outline and mixed medium to slightly high signal intensity in the anterior horn of the lateral meniscus on sagittal T1WI and PDWI-FS sequences. The T1WI displays moderate patchy signals within the meniscus, while the PDWI-FS sequence indicates slightly elevated patchy T2 signals (red arrows), with unclear boundaries. These abnormal signals extend to the meniscus surface. Arthroscopy (c) confirms MSU crystal deposition on the surface of the lateral meniscus (red arrow). The patient is a 46-year-old man with a 7-year history of KGA (d–f). The sagittal T1WI sequence (d) reveals an irregular nodular-shaped patch with moderate signal intensity on the surface of the anterior horn of the lateral meniscus (red arrow). The sagittal and axial PDWI-FS sequences (e, f) show irregular patches with slightly elevated T2 signal intensity (red arrows), with clear boundaries and a normal meniscus morphology.

In most patients with urate crystal deposits in the meniscus, MRI shows a patchy or diffuse medium to slightly high T1 and T2 signals within the meniscus, with unclear borders (Fig. 4 a, b). Although the overall morphology of the meniscus generally appears normal, arthroscopy identifies urate crystal deposits on the meniscal surface without signs of degeneration or damage (Fig. 4 c). In fewer cases, MRI directly visualizes urate crystal deposition on the meniscus surface as small, round lesions with moderate to slightly elevated T1 and T2 signals (Fig. 4 d-f). The morphology of the meniscus usually remains unaffected.

**Fig. 5:**
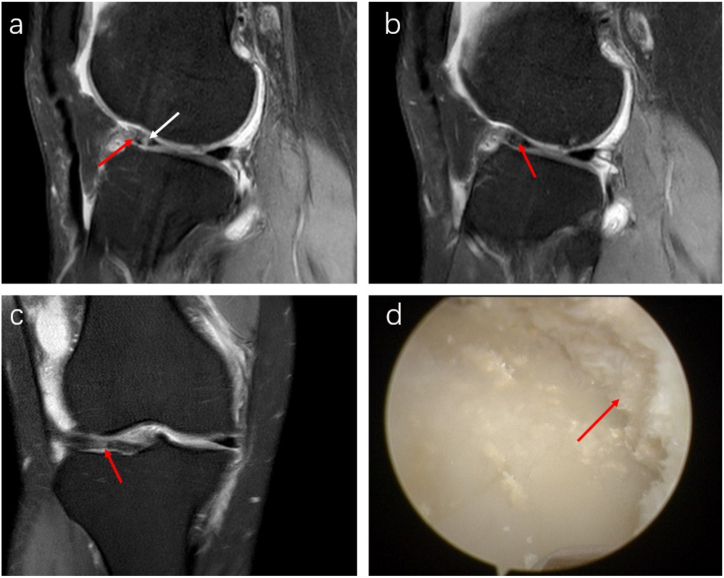
MRI and arthroscopic images of KGA combined with meniscus tear. A 34-year-old male patient with gout and a lateral meniscus tear was examined. Sagittal (a, b) and coronal (c) PDWI-FS sequence images revealed a radial tear (indicated by a white arrow) in the anterior horn of the lateral meniscus. Additionally, an abnormal, moderately to highly intense T2 signal within the meniscus (indicated by a red arrow) displayed unclear borders. Arthroscopic examination (d) showed damage to the anterior horn of the lateral meniscus and deposition of MSU crystals (indicated by a red arrow).

In patients with KGA concurrent with meniscal tears, sagittal and coronal PDWI-FS sequences reveal high T2 signal intensity abnormal tears, meniscal defects or displacements, and mixed slightly high-to-medium signal intensity within the meniscus (Fig. 5 a-c). Arthroscopy confirmed damage to the anterior horn of the lateral meniscus and deposition of MSU crystals (Fig. 5 d).

### 3.6 Cartilage irregularly serrated changes

**Fig. 6:**
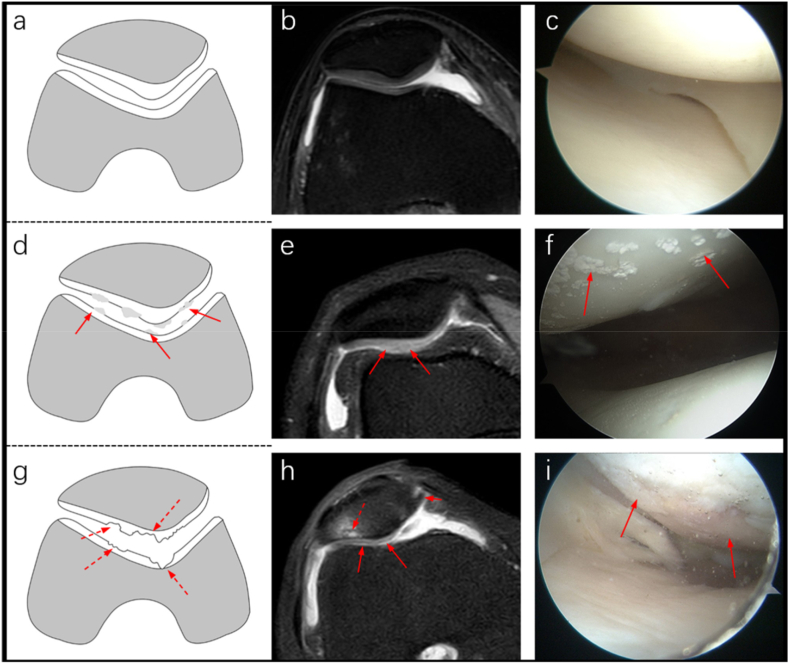
Schematic diagrams, along with MRI and arthroscopic images, depicted the patellofemoral cartilage of normal individuals, KGA patients, and osteoarthritis patients. Diagrams a-c represent healthy adults; d-f depict knee gouty arthritis, with urate deposits visible on or infiltrating the cartilage surface (indicated by solid red line arrows). Axial PDWI-FS sequence (e) showed irregular cartilage contours with a sawtooth appearance (indicated by red arrows) and increased cartilage thickness. Arthroscopic examination (f) confirmed urate crystal deposition on the patellar cartilage (indicated by red arrows). Diagrams g-i represent osteoarthritis. The schematic diagram (g) showed visible cartilage defects (indicated by red dashed line arrows). Axial PDWI-FS sequence (h) displayed widespread patellar cartilage defects with significant thinning, showing crater-like alterations (indicated by red solid line arrows). Additionally, narrow joint spaces, patchy high T2 signals in the subchondral bone (indicated by red dashed line arrows), and medial patellar bone spurs (indicated by short red solid line arrows) were observed.

The study indicated that urate crystal deposits on cartilage surfaces often cause irregularities in the cartilage contour on PDWI-FS sequences, displaying serrated changes. The signal intensity might be normal or elevated, and the cartilage thickness might remain unchanged or increase. Moderate to slightly elevated T2 signal intensity was observed in the cartilage (Fig. 6 d, e). Arthroscopy revealed either scattered or diffuse deposition of MSU crystals on the cartilage surface (Fig. 6 f). In contrast, osteoarthritis typically presents as roughening on the cartilage surface with localized thinning or complete loss of layers on MRI, showing crater-like alterations (Fig. 6g and h). Arthroscopic examination revealed cartilage softening, defects, and exposed subchondral bone (Fig. 6 i). These findings highlight the differences between the two conditions.

**Fig. 7:**
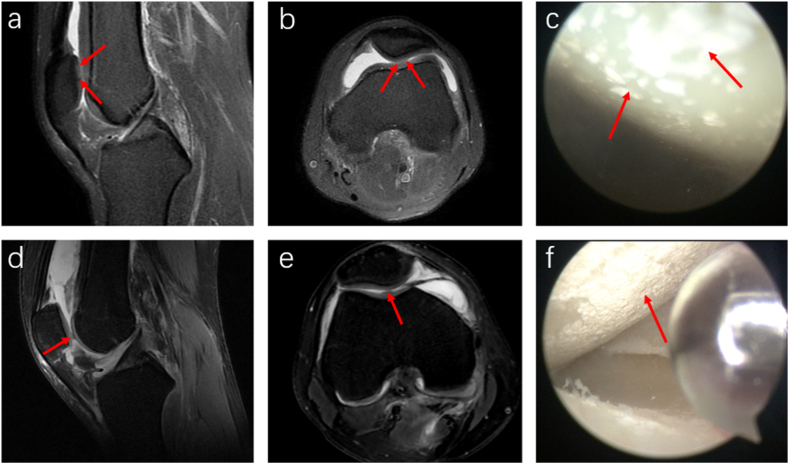
MRI findings and arthroscopic imaging of urate deposition in KGA cartilage. Direct MR images demonstrated MSU crystal deposition on the cartilage surface (a, b, d, e). In the sagittal (a, d) and axial (b, e) PDWI-FS sequences, deposition of MSU crystals on the femoral trochlear cartilage surface was observed (indicated by red arrows), presenting as areas of irregular moderate to slightly high T2 signal intensity with clear boundaries. There was significant fluid accumulation in the suprapatellar bursa, and patchy slightly high T2 signal intensity areas were visible within the infrapatellar fat pad. Arthroscopic examination (c, f) revealed diffuse MSU crystal deposits on the femoral trochlear cartilage surface (indicated by red arrows).

In some cases, MRI may detect amorphous aggregates of MSU crystals on the cartilage surface, displaying moderate to slightly high T1 and T2 signal intensities (Fig. 7 a, b, d, e). Arthroscopic examination confirmed the presence of MSU crystal deposits on the cartilage surface (Fig. 7 c, f).

### 3.7 MRI features of synovitis

**Fig. 8:**
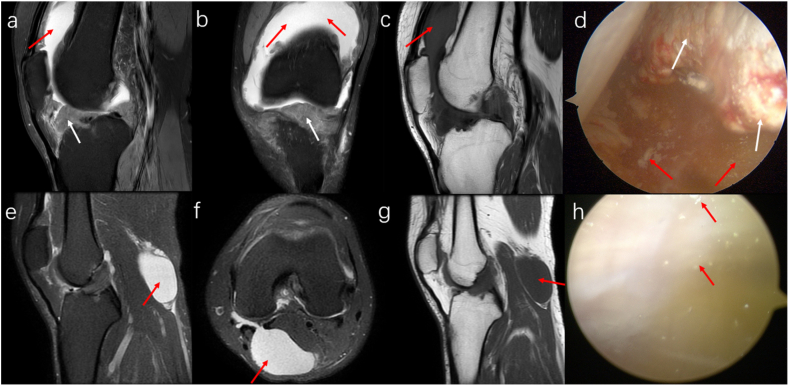
MRI and arthroscopic images of joint effusion, synovial proliferation, and popliteal cysts. A 33-year-old male with a 14-year history of KGA exhibits significant joint effusion in the suprapatellar bursa on sagittal (a) and coronal (b) PDWI-FS sequences, marked by strip-like high T2 signal intensity areas. These include diffusely low T2 signal intensity areas (red arrows), resembling the ultrasound's snowstorm sign. Sagittal T1WI sequence (c) shows slightly low T1 signal intensity (red arrows). Extensive synovial hyperplasia within the joint cavity is noted (white arrows), appearing as low T1 and moderate to slightly low T2 signal intensity (a–c). Arthroscopic examination (d) confirms synovial hyperplasia (white arrows) and intra-articular MSU crystal deposition (red arrows). Another patient, a 30-year-old male with a 4-year history of KGA (e–h), exhibits a popliteal cyst behind the knee on sagittal and axial PDWI-FS sequences (e, f; red arrow), showing high T2 fluid signal intensity. Low T2 signal intensity areas within the cyst are not clearly visible on the T1WI sequence (g). Arthroscopy (h) reveals MSU crystal deposition within the cyst (red arrow).

PDWI-FS sequences reveal varying degrees of low T2 signal intensity within joint effusions, which appear as strip-like low T1 and moderate to high T2 signal intensity areas on MRI. This study identified irregular low T2 signal intensity areas within high signal intensity effusions on PDWI-FS sequences. These areas were distributed unevenly and displayed low or slightly low T1 signal intensity, blending indistinctly with the surrounding joint effusion (Fig. 8 a-c). Arthroscopic examination confirmed MSU crystal deposition in the joint fluid (Fig. 8 d). Excessive joint effusion can lead to a popliteal cyst, which may show similar MRI characteristics (Fig. 8 e-g). Urate crystals within the cyst were also confirmed by arthroscopy (Fig. 8 h).

Synovial hyperplasia in KGA patients often occurs around the cruciate ligaments, fat pad, and tendon sheaths. The hyperplasia typically has an irregular shape with unclear boundaries and displays moderate to slightly low T2 signal intensity on MRI. In cases of severe knee involvement, sagittal MRI might show the meniscus surrounded by proliferative synovium (Fig. 8 a-c), indicating abnormal meniscal morphology, whereas coronal MRI usually appears normal. This condition helps differentiate meniscal tears from other anomalies using multi-angle MRI. Arthroscopic findings included synovial hyperplasia in the intercondylar fossa (Fig. 8 d).

Hoffa's fat pad synovitis manifests as nodular or patchy low or slightly low T1 signals within the infrapatellar fat pad on T1WI and slightly high T2 signals on PDWI-FS sequence. In severe cases, the fat pad might appear completely disorganized, presenting a diffuse high T2 signal (Supplementary Fig. S2).

### 3.8 MRI features of gouty tophi, bone erosion, and bone marrow edema

Gouty tophi appear in various shapes on MRI, such as linear, nodular, or mass-like formations. They are commonly found around the lateral femoral condyle's peritendinous area, the suprapatellar pouch, the infrapatellar fat pad, and the intercondylar fossa. On T1WI, they exhibit a moderately heterogeneous or slightly low signal. On the PDWI-FS (Supplementary Fig. S3 a, b), they show a moderately heterogeneous or slightly high signal.

Bone erosion is often seen on the lateral femoral condyle. On T1WI, circular or oval-shaped bone defects are visible with sharp margins and a limited extent. In some patients with severe involvement of the knee joint tissues, the “overhanging edge sign” is observed, characterized by mild cortical expansion or localized defects near the bone destruction area, creating a raised edge (Supplementary Fig. S3 c, d).

Bone marrow edema is commonly found in the tibiofemoral joint, usually near gouty tophi or bone erosions, but is generally limited in extent. Typically, the edema does not exceed 5 mm in depth, and the borders are well-defined. On T1WI, it shows a moderate or slightly low signal, while on PDWI-FS, it presents a high T2 signal (Supplementary Fig. S3 c, d).

### 3.9 Test-retest reliability of the MRI features

The ICC values for various MRI features, such as ligament changes, meniscal urate deposition, and cartilage irregularities, which were 0.913, 0.670, 0.793, 0.875, 0.783, 0.760, 0.801, and 0.913, respectively, indicating good to excellent reliability between Reader 1 and Reader 2. Specific ICC values included 0.738 for ligament changes and 0.913 for both meniscal urate deposition and bone marrow edema. These findings are detailed in Table 2.

**Table 2:** Intraclass correlation coefficients for interobserver test-retest reliability (n = 49) of the MRI features.

MRI features (n = 49)	ICC	95％CI	P value
Ligament sketch-like changes	0.738	0.579, 0.843	***
ACL	0.833	0.721, 0.903	***
PCL	0.916	0.856, 0.952	***
LCL	0.640	0.436, 0.780	***
MCL	0.854	0.755, 0.915	***
Meniscal urate deposition changes	0.913	0.851, 0.950	***
MMAH	0.755	0.599,0.855	***
MMB	0.676	0.487, 0.804	***
MMPH	0.640	0.436, 0.780	***
LMAH	0.693	0.515, 0.814	***
LMB	0.706	0.524, 0.825	***
LMPH	0.706	0.534, 0.823	***
Cartilage irregularly serrated changes	0.670	0.490, 0.807	***
PJ	0.686	0.470, 0.818	***
TJ	0.458	0.208, 0.652	***
Point or diffuse low-signal signs within Joint effusion	0.793	0.661, 0.878	***
Synovial proliferation	0.875	0.787, 0.928	***
Hoffa's fat pad synovitis	0.783	0.647, 0.871	***
Gouty tophi	0.760	0.594, 0.861	***
Bone erosion	0.801	0.663,0.855	***
Bone marrow edema	0.913	0.850, 0.950	***

ACL, anterior cruciate ligament; PCL, posterior cruciate ligament; LCL, lateral collateral ligament; MCL, medial collateral ligament; MMAH, medial meniscus anterior horn; MMB, medial meniscus body; MMPH, medial meniscus posterior horn; LMAH, lateral meniscus anterior horn; LMB, lateral meniscus body; LMPH, lateral meniscus posterior horn; PJ, patellofemoral joint; TJ, tibiofemoral joint; ICC, intraclass correlation coefficient; CI, confidence interval. ***P < 0.001.

### 3.10 Comparison of MRI features between the two groups of patients

In the severe group, the occurrence rates of ACL changes, Hoffa's fat pad synovitis, and gouty tophi were significantly higher compared to the mild group (P < 0.05). However, no significant differences were observed in meniscal urate deposition, cartilage changes, joint effusion signals, bone erosion, or bone marrow edema between the two groups (P > 0.05). These results are summarized in Table 3.

**Table 3:** Comparison of MRI features between the mild and severe KGA groups.

MRI features	Mild group (n = 18)	Severe group (n = 31)	P value
Ligament sketch-like changes	13, 72.2％	30, 96.8％	*
ACL	10, 55.6％	26, 83.9％	*
PCL	5, 27.8％	13, 41.9％	–
LCL	9, 50.0％	21, 67.7％	–
MCL	2, 11.1％	5, 16.1％	–
Meniscal urate deposition changes	15, 83.3％	27, 87.1％	–
MMAH	4, 22.2％	8, 25.8％	–
MMB	4, 22.2％	5, 16.1％	–
MMPH	7, 38.9％	6, 19.4％	–
LMAH	11, 61.1％	23, 74.2％	–
LMB	3, 16.7％	10, 32.3％	–
LMPH	4, 22.2％	3, 9.7％	–
Cartilage irregularly serrated changes	9, 50.0％	15, 48.4％	–
PJ	7, 38.9％	14, 45.2％	–
TJ	3, 16.7％	3, 9.7％	–
Point or diffuse low-signal signs within the joint effusion	17, 94.4％	30, 96.8％	–
Synovial proliferation	9, 50.0％	19, 61.3％	–
Hoffa's fat pad synovitis	10, 55.6％	26, 83.9％	*
Gouty tophi	4, 22.2％	20, 64.5％	*
Bone erosion	6, 33.3％	16, 51.6％	–
Bone marrow edema	15, 83.3％	27, 87.1％	–

*P < 0.05. “-” means P is greater than 0.05.

### 3.11 Analysis of the common sites of urate deposition in ligaments, menisci, and cartilage

**Fig. 9:**
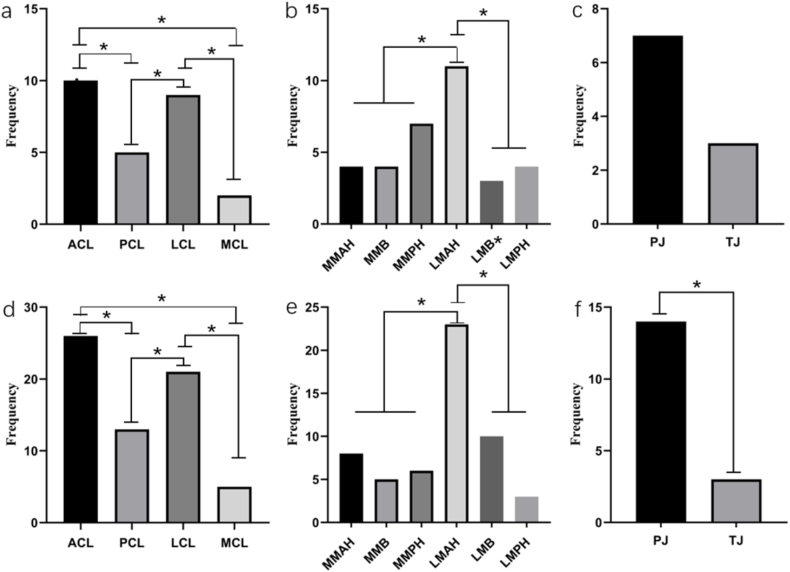
Chi-square goodness-of-fit analysis of the common sites of urate deposition in the ligaments, menisci, and cartilage. Ligament, meniscus, and cartilage in the mild group (a–c); ligament, meniscus, and cartilage in the severe group (d–f). ACL, anterior cruciate ligament; PCL, posterior cruciate ligament; LCL, lateral collateral ligament; MCL, medial collateral ligament; MMAH, medial meniscus anterior horn; MMB, medial meniscus body; MMPH, medial meniscus posterior horn; LMAH, lateral meniscus anterior horn; LMB, lateral meniscus body; LMPH, lateral meniscus posterior horn; PJ, patellofemoral joint; TJ, tibiofemoral joint. “*" means P ＜0.05.

Research findings show that ligament changes resembling sketches are most common in the ACL, followed by the LCL, while the PCL and MCL show fewer changes; these differences are statistically significant (Fig. 9 a, d). In the mild group and severe group, urate deposits in the meniscus were more prevalent in the anterior horn of the lateral meniscus, the differences are statistically significant (Fig. 9 b, e). No significant difference was observed in the distribution of irregularly serrated cartilage changes in the mild group, but in the severe group, changes were more common in the patellofemoral joint than in the tibiofemoral joint, the differences are statistically significant (Fig. 9 c, f).

### 3.12 Correlation analysis of the degree of tissue involvement in KGA

Cramer's V coefficients indicated significant positive correlations between ACL sketch-like changes and tissue involvement in the knee joint assessment (r = 0.309, P < 0.05). Hoffa's fat pad synovitis and gouty tophi also showed positive correlations with tissue involvement (r = 0.309 and r = 0.408 respectively; P < 0.05 and P < 0.01).

### 3.13 Multivariate logistic regression analysis of the degree of tissue involvement in KGA

Logistic regression analysis was performed to assess the degree of tissue involvement in patients with KGA, classifying it as either mild (1) or severe (2). Variables such as disease duration, serum uric acid concentration, BMI, and MRI features such as ACL sketch-like changes, Hoffa's fat pad synovitis, and gouty tophi were included. The analysis indicated that ACL changes (OR = 9.019, 95 % CI = 1.364–61.880; P < 0.05), Hoffa's fat pad synovitis (OR = 6.472, 95 % CI = 1.041–40.229; P < 0.05), and gouty tophi (OR = 5.972, 95 % CI = 1.218–29.276; P < 0.05) are independent risk factors for predicting the degree of tissue involvement in KGA patients (Table 4).

**Table 4:** Multivariable logistic regression analysis of the extent of KGA tissue involvement.

Factors	Regression coefficients	Standard error	Waid χ^2^ value	P value	OR	95％ CI
Age	0.023	0.050	0.219	–	1.002	[0.929–1.128]
Disease duration	−0.005	0.007	0.477	–	0.995	[0.981–1.009]
BMI	−0.010	0.098	0.011	–	1.010	[0.835–1.223]
UA	0.006	0.004	3.204	–	1.006	[0.999–1.014]
ACL sketch-like changes	2.199	0.983	5.010	*	9.019	[1.364–61.880]
Hoffa's fat pad synovitis	1.868	0.932	4.014	*	6.472	[1.041–40.229]
Gouty tophi	1.787	0.811	4.854	*	5.972	[1.218–29.276]

OR, odds ratio. *P < 0.05. “-" means P is greater than 0.05.

### 3.14 Analysis of the predictive value of the degree of tissue involvement in KGA

**Fig. 10:**
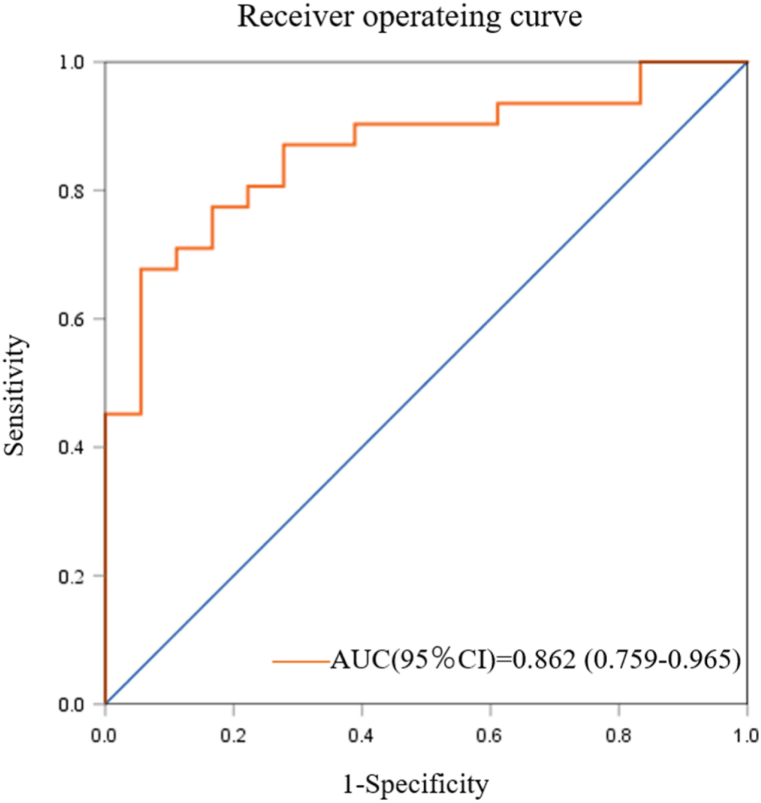
ROC curve prediction of severe tissue involvement in KGA patients. Combined prediction based on ACL sketch-like changes, Hoffa's fat pad synovitis, and gouty tophi.

The ROC curve analysis (Fig. 10) demonstrated that ACL sketch-like changes, Hoffa's fat pad synovitis, and gouty tophi can predict severe tissue involvement, with an area under the curve of 0.862 [95 % CI (0.759, 0.965)]. The optimal cutoff value of 0.622 provided a sensitivity of 67.70 %, specificity of 94.40 %, and accuracy of 79.14 %.

## 4 Discussion

Gout is an increasingly prevalent and challenging condition to manage. Its prevalence in China ranges from 1 % to 3 % and has been increasing in recent years [4,7]. Although gout typically begins between the ages of 40 and 50 years, recent studies indicate a shift toward younger individuals [25]. In our research, we focused on KGA patients. Remarkably, 85.7 % of these patients were under 40 years old, with the youngest being only 17. These findings confirm a significant trend toward earlier onset of KGA. Given KGA's high prevalence and the complexity of its management—coupled with a lack of comprehensive assessments using MRI for tissue evaluation—we recognized a gap in research. This study aims to bridge that gap by providing valuable insights into the impact of KGA on different knee tissues using MRI. The interobserver intraclass correlation coefficients for MRI features were strong, indicating high reproducibility and underscoring the crucial role of MRI in diagnosing KGA.

This study identified various MRI features in the knee joints of patients with KGA, such as ligament sketch-like changes, meniscal urate deposition changes, cartilage irregularly serrated changes, point or diffuse low-signal signs within joint effusion, gouty tophi, and bone erosion. Notably, ligament sketch-like changes, meniscal urate deposition changes, and point or diffuse low-signal signs within joint effusion were recognized as characteristic MRI features for diagnosing KGA. Cartilage irregularly serrated changes could aid in differentiating KGA in young and middle-aged patients. Gouty tophi and bone erosion are typical of late-stage manifestations of KGA. However, findings such as bone marrow edema and synovial proliferation have shown limited diagnostic value. Furthermore, ACL sketch-like changes, Hoffa's fat pad synovitis, and gouty tophi correlate positively with tissue severity and can independently predict the extent of tissue damage in KGA.

Current research on MRI imaging of ligaments with urate crystal deposition is limited. Earlier studies indicate that accumulation of MSU crystals in ligaments and tendon attachment points can lead to fibrous degeneration [26]and reduced tensile strength, potentially causing ligament rupture after minor trauma [[27], [28], [29]]. In the initial stages of this study, some KGA patients showed MRI signals in the ACL resembling ligament injuries, termed sketch-like changes. This is suspected to be closely linked to MSU crystal deposition. Arthroscopic examination confirmed this correlation, and further observations indicated the prevalence of these MRI findings in ligaments affected by MSU crystals, particularly in the ACL. This difference might be due to the synovial characteristics of the ACL's surface. Thus, this MRI feature, common in mild KGA cases, may be a useful diagnostic marker.

When the ligament is severely damaged, it often presents as diffuse patches with medium to high signal intensity. The fibers become disorganized, resembling the tendrils of a monocotyledonous plant, leading to what may be termed a “fibrous root-like” ACL. Distinguishing between KGA and ACL injuries in clinical practice is crucial for appropriate management. A definitive diagnosis is typically made by considering the patient's medical history, including any trauma or history of gout, along with clinical tests such as drawer tests, Lachman tests, and pivot-shift tests, and MRI findings that show sketch-like changes in the ACL.

Meniscal urate deposition is another MRI feature indicative of KGA. A case report highlighted by DECT showed urate deposits within the meniscus [30], though it was unclear if the meniscus itself was injured. Our study also found MRI evidence of MSU crystal deposition in the meniscus, characterized by patchy or diffuse medium to slightly high T1 and T2 signal lesions on T1 and T2, and nodular or globular lesions on PDWI-FS sequences. Arthroscopic confirmation showed these were not related to meniscal degeneration or injury.

In clinical observations, patients with KGA who had urate deposits on or within the meniscus often experienced meniscal injuries. It is believed that urate deposition may change the mechanical properties of the meniscus, increasing injury risk. Studies, such as those by CAMPO-RUIZ et al. [31], have shown that MSU crystals can compress and erode the meniscus, leading to destruction of the meniscal matrix and abnormal vascular growth, suggesting that these signal changes may be due to MSU crystal erosion. This supports the hypothesis that urate deposition could initiate meniscal injury. However, other studies suggest that collagen may play an important role in urate nucleation, accelerating the deposition of MSU crystals. CHHANA and XU et al. [32,33] reported that type II collagen promotes urate deposition on the cartilage surface. While there is no current research specifically on the meniscus, it is suspected that exposure of the meniscus's internal collagen due to injury could also promote MSU crystal deposition. Nonetheless, the exact relationship between meniscal injury and urate deposition requires further investigation.

MSU crystals typically deposit on the surface of knee joint cartilage, causing compression and erosion of the surrounding cartilage [34,35]. Zhang, Duan et al. [36]demonstrated that UA can be transported into the chondrocyte cytoplasm through GLUT9 and URAT1, leading to cartilage lesions. Both the mechanical abrasion from urate crystals and the immune response associated with gout can damage cartilage, potentially leading to secondary osteoarthritis [32,37]. Therefore, distinguishing between KGA and osteoarthritis is crucial. In the present study, MSU crystals were commonly found in the patellofemoral joint, particularly along the patellar ridge, likely due to increased stress in this area which promotes urate deposition [38,39]. We also observed that cartilage in KGA patients often appears with an irregular serrated signal change on MRI, similar to cartilage defects seen in osteoarthritis [3,40]. However, MRI findings of cartilage differ between the two diseases; osteoarthritis typically shows thinned or absent joint cartilage, subchondral bone edema, sclerosis, cystic changes, and osteophyte formation [41,42], while KGA patients usually have normal or increased cartilage thickness with localized lesions that do not involve the subchondral bone.

In some cases of KGA, MRI may show irregularly shaped medium or slightly elevated T1 and T2 signal MSU aggregates on the cartilage surface. Arthroscopic examinations in KGA patients typically reveal scattered or widespread urate crystal deposition on the cartilage surface without cartilage tears or exposed subchondral bone. These findings suggest imaging evidence of MSU crystal deposition or erosion (Supplementary Table S1). Notably, as the onset age of KGA decreases, it is becoming more common among young and middle-aged adults, whereas osteoarthritis predominantly affects middle-aged and elderly individuals [43,44]. This study suggests that changes in cartilage urate deposition may be valuable for the differential diagnosis of KGA in young and middle-aged patients.

KGA disease progression is significantly influenced by the deposition of MSU crystals in the joints, which is critical for the onset of the disease. Even in the early, asymptomatic stages, MSU [45] crystals may begin to accumulate in the joints. This deposition triggers microscopic synovial inflammation, leading to synovial thickening, adipose tissue inflammation, and the formation of joint effusion [15,46].

Studies have shown that urate crystal deposits can appear as lower signal intensities on MRI scans. Validated by arthroscopy, this research confirmed that joint effusions or popliteal cysts in KGA patients exhibiting diffuse low signals are similar in diagnostic significance to the “punctate hyperechoic sign” seen in gout ultrasonography [34,37]. These MRI findings can indicate MSU crystal presence in joint fluid, serving as a distinct feature for identifying KGA. Arthroscopic evidence further supports that MSU crystals in joint effusions or popliteal cysts can present as point or diffuse low signals in the PDWI-FS MRI sequence. This is akin to the point hyperechoic or snowstorm signs seen in ultrasound. While ultrasound [7,30,47] allows for dynamic exploration, MRI provides static images, which is a slight limitation. However, these MRI signs are indicative of MSU crystal deposition and are essential for diagnosing and distinguishing KGA.

Hoffa's fat pad synovitis primarily arises from trauma and inflammation, leading to the swelling, proliferation, or enlargement of the fat pad [48,49]. On PDWI-FS sequences, it is visible as localized or diffuse high signal intensity. The study indicates that Hoffa's fat pad is a frequent site of urate crystal accumulation in KGA patients, which promotes the release of inflammatory factors such as IL-1β, triggering an inflammatory response [[50], [51], [52], [53]]. Hence, Hoffa's fat pad synovitis serves as an indicator of the extent of inflammation within knee joint tissues. The incidence of Hoffa's fat pad synovitis is notably higher in the severe group than in the mild group (P < 0.05), suggesting a correlation with the progression of KGA and highlighting its significance in assessing the degree of knee joint tissue involvement.

Gouty tophi and bone erosion are key MRI features of chronic KGA and are closely associated with the presence of MSU crystals. Gouty tophi can trigger inflammatory reactions in joints, accelerating the progression of KGA [54]. Steinmetz et al. found that using arthroscopic minimally invasive techniques to remove gouty tophi or MSU crystals from joints can significantly delay the progression of KGA and improve prognosis in patients with severe intra-articular tissue involvement [55]. This study frequently observed gouty tophi in knee joints with extensive tissue involvement, typically presenting as intermediate to slightly high mixed T2 signals. Wang et al. suggested that bone destruction is closely linked to the compression and destruction of bone by gout tophi [56]. Bone erosion was commonly found adjacent to gouty tophi, and some KGA patients displayed the characteristic “rim sign” of gouty tophi accompanied by bone erosion [[57], [58], [59]]. In this study, bone erosion was more prevalent on the lateral condyle of the femur, possibly due to calcification at the origin of the popliteal tendon or the presence of gouty tophi.

Previously, factors such as serum uric acid levels, disease duration, and BMI were thought to contribute to the progression of KGA. Therefore, this study included clinical indicators such as serum uric acid levels, disease duration, and BMI in a multiple regression analysis. However, the results showed no statistically significant differences, indicating that compared to MRI features, factors like age, KGA disease status, BMI, and serum uric acid levels have limited predictive value for the degree of tissue involvement or disease progression in KGA patients.

### 4.1 Limitations of this study

The primary limitation of this study was that it was a single-center, cross-sectional study with a small sample size. The participants were mainly individuals undergoing arthroscopic treatment, which may introduce selection bias. Future studies should expand to multiple centers, increase the sample size, and include detailed postoperative follow-ups to enhance the reliability and generalizability of the findings. Prospective and longitudinal studies are needed to validate MRI features that can predict or diagnose KGA and to explore their relationship with clinical progression and treatment responses. Additionally, this study relied on standard MRI scans, which may not be sensitive enough to detect cartilage MSU crystal deposition. Future research should incorporate advanced MRI sequences, such as T2 mapping MRI, which can detect early biochemical changes in cartilage and potentially enable early detection of cartilage damage in KGA patients.

## 5 Conclusion

In conclusion, specific MRI findings such as ligament sketch-like changes, meniscal urate deposition, and low-signal signs in joint effusion are characteristic indicators for diagnosing KGA. Furthermore, irregular cartilage changes are diagnostically valuable in young and middle-aged patients. Our study found that ACL sketch-like changes, Hoffa's fat pad synovitis, and gouty tophi are linked to the severity of tissue involvement in KGA. These features are significant predictors of the extent of tissue involvement and can guide clinical diagnosis and treatment, ultimately improving patient outcomes.

## Ethics declarations

This was a retrospective study. The study obtained approval from the Medical Ethics Committee of the Second Hospital of Jilin University, and the requirement for informed consent from the subjects was waived (approval number: 2023 Research Review NO. 2023136).

## Consent for publication

Not applicable.

## Availability of data and materials

The data that support the findings of this study are not openly available due to reasons of sensitivity. Data included in article/supp. material/referenced in article. The data were obtained via controlled access data storage at Jilin University Second Hospital and are available from the corresponding author upon reasonable request.

## Funding

This research was funded by the 10.13039/100006180Jilin Provincial Science and Technology Development Plan Key R&D Fund (20240305034YY).

## CRediT authorship contribution statement

**Qingshuai Wang:** Writing – original draft, Visualization, Conceptualization. **Bo Chen:** Formal analysis. **Zhicheng Zhang:** Investigation. **Xiongfeng Tang:** Writing – review & editing, Methodology, Conceptualization. **Yingzhi Li:** Validation, Supervision, Resources, Conceptualization.

## Declaration of competing interest

The authors declare that they have no known competing financial interests or personal relationships that could have appeared to influence the work reported in this paper.
