# Supplementary material for: Correlations of characteristics with tissue involvement in knee gouty arthritis: Magnetic resonance imaging analysis
Source: Heliyon. 2024 May 23;10(11):e31888. doi: 10.1016/j.heliyon.2024.e31888 (PMC11152737; doi:10.1016/j.heliyon.2024.e31888)
Supplement: Multimedia component 1 [file mmc1.docx]

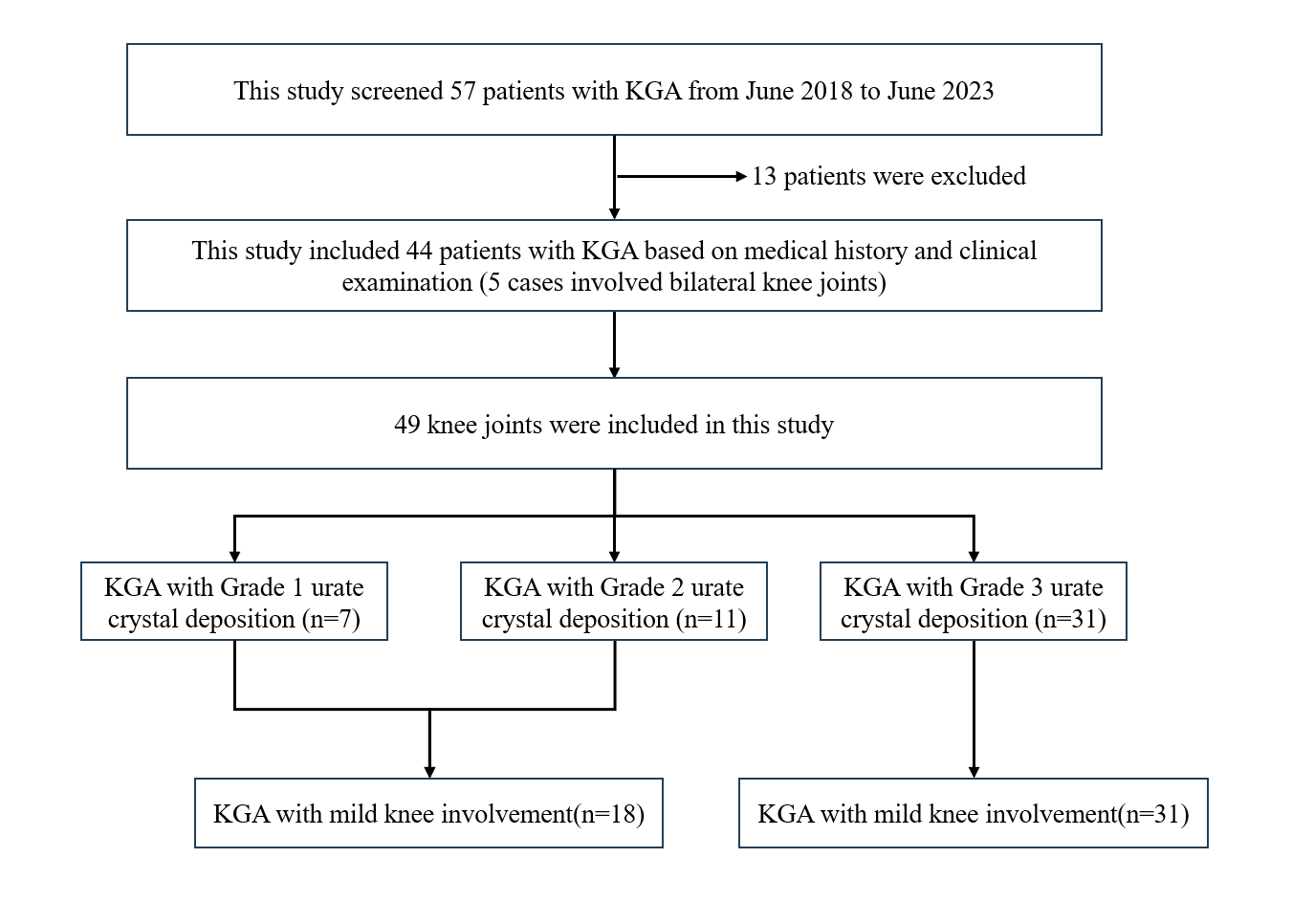


**Supplementary Fig. S1** Flowchart for KGA participating in the study.


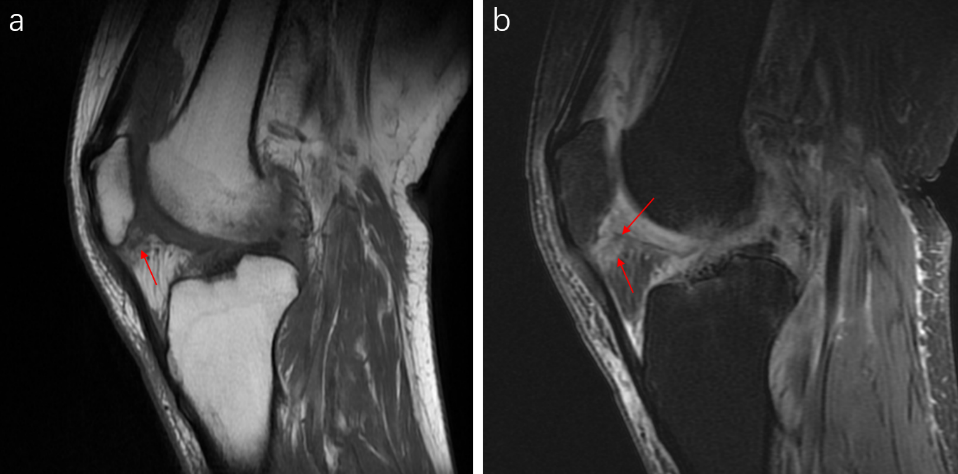


**Supplementary Fig. S2** MRI Manifestations of Hoffa's fat pad synovitis. In the sagittal T1-weighted sequence (a), patchy long T1 signals are observed within the infrapatellar fat pad(red arrow). The PDWI-FS sequence (b) reveals complete disarray of the infrapatellar fat pad, presenting as diffuse elevated T2 signals (red arrow).


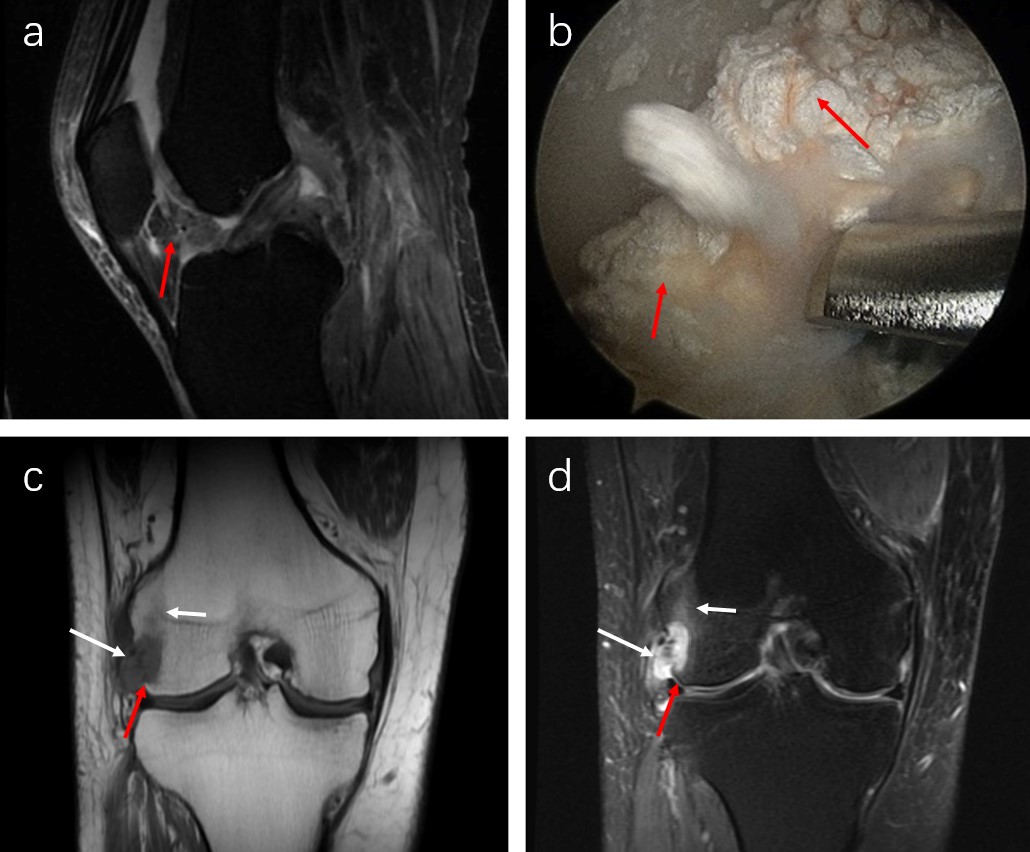


**Supplementary Fig. S3** MRI images of gouty tophi, bone erosion, and bone marrow edema. In the sagittal PDWI-FS sequence (a), there is a mass-like tophaceous gout near the intercondylar notch (red arrow). The nodules exhibit medium or slightly lower mixed signal intensity. The ACL appears irregularly thickened with heterogeneous signal intensity, showing irregular patchy slightly high signals. Under arthroscopic examination (b), irregular gouty tophus nodules are visible (red arrow), located near the intercondylar notch. In coronal T1-weighted imaging (c) and PDWI-FS sequence (d) of the left knee, MRI reveals the presence of an irregular gouty tophus surrounding the lateral condyle of the right femur (white long arrow). On T1WI, the tophus appears with a uniform low signal, while on PDWI-FS sequence, it displays a mixed intermediate-to-high signal. Additionally, adjacent to the lateral condyle of the femur, there is circular punched-out bone destruction and the “overhanging edge sign” (red arrow). Furthermore, a nearby, patchy bone marrow edema signal is also visible (white short arrow).

**Supplementary Table S1** Differential diagnosis between Osteoarthritis and KGA

|  | **Osteoarthritis**^[1-4]^ | **Knee gouty arthritis** |
| --- | --- | --- |
| Age | Middle-aged and elderly | young adults |
| Cartilage MRI signal | Normal signal or increased T2 signal | normal internal signal or increased T2 signal; moderately or slightly increased T1 and T2 signals on the cartilage surface |
| Cartilage thickness | Partial thinning or full layer disappearance | normal or thickened |
| Cartilage contour | irregular or defective | irregularities in the cartilage contour, displaying jagged changes |
| Subchondral bone | Bone edema, sclerosis, or cystic degeneration | normal |
| Joint space | usually narrow | usually normal |
| Osteophytes | usually exist | usually none |
| Bone erosion | none | usually exist |
| Arthroscopic findings | Cartilage swelling, tearing, or exposure of subchondral bone | Scattered or diffuse deposition of MSU crystals on cartilage surface |

[1] CAO L, ZHAO T, XIE C, et al. Performance of Ultrasound in the Clinical Evaluation of Gout and Hyperuricemia [J]. Journal of Immunology Research, 2021, 2021 <https://doi.org/10.1155/2021/5550626>.

[2] TAN Y K, CONAGHAN P G. Insights into osteoarthritis from MRI [J]. International Journal of Rheumatic Diseases, 2012, 15(1): 1-7. <https://doi.org/10.1111/j.1756-185X.2011.01677.x>.

[3] RODRIGUES M B, CAMANHO G L. MRI EVALUATION OF KNEE CARTILAGE [J]. Revista brasileira de ortopedia, 2010, 45(4): 340-6. <https://doi.org/10.1016/s2255-4971(15)30379-7>.

[4] PETERFY C G, GUERMAZI A, ZAIM S, et al. Whole-organ magnetic resonance imaging score (WORMS) of the knee in osteoarthritis [J]. Osteoarthritis and Cartilage, 2004, 12(3): 177-90. <https://doi.org/10.1016/j.joca.2003.11.003>.
